# Sense-making as digitalization. Measuring, counting, and calculating as fundamental processes of digital ‘Bildung’

**DOI:** 10.3389/fsoc.2025.1552621

**Published:** 2025-12-03

**Authors:** Sebastian Manhart

**Affiliations:** 1 Institut für Bildungswissenschaft, Fakultät für Humanwissenschaften, Universität der Bundeswehr München, Neubiberg, Germany; 2 Institute of Educational Sciences, The Department of Human Sciences, Munich University of the Federal Armed Forces, Neubiberg, Germany

**Keywords:** sense-making, semiosis, information, calculation, measurement, communication, digtalization, artificial intelligence

## Abstract

The article provides a clarification of the concept of digitalization, bringing together its two dimensions: electronic-physical information transformation and the social aspect of connection through the sense-making and creation of meaning via numbers. This is done both conceptually and by presenting the historical development of this socially complex synthesis of causality, sense-making and meaning. Fundamental aspects of social change from communication to information processing, from facts to data, from true nature to virtual possibility, from face-to-face sociality to digital habituation, from human to digital intelligence are presented in their development using historical examples. The focus is on the practices of counting, calculating, and measuring, whose gradual differentiation from communication enables the unlikely process of social implementation of an operationally meaningful but at the same time completely reference-less (digital) semiosis. The analysis of these specifically modern organizational and pedagogical developments as a prerequisite for digitization allows conclusions to be drawn about some aspects of digital subjectivity and society in the age of artificial intelligence.

## 1 Introduction

Many changes in the present are understood as effects of digitalization. Digitalization not only influences the production and distribution conditions of goods and services in industry (Acemoglu and Restrepo, 2018, 2020; Altenried, 2022) but also in agriculture (Rijnks et al., 2022) and on the labor markets (Gomez-Herrera et al., 2023); it changes decision-making behavior (Wendt and Manhart, 2020) and the forms and content of interpersonal communication (Kelkar, 2018). In legal (Beckers and Teubner, 2021), policing (Büchner and Dosdall, 2021), organizational (Manhart and Wendt, 2021), or educational contexts (Hartong, 2019; Wendt and Manhart, 2022; Aßmann and Ricken, 2023; Buck and Miguel, 2023; Shin et al., 2025; Wang and Fan, 2025), there are also numerous examples of fields of action and influence that are being changed by digitalization. The effects of digitalization are not only very different across sectors, regions, and cultures but also affect genders in very different ways (del Egana- Sol et al., 2021). Euphoric expectations regarding economic (Hackl and Buzzle, 2021) and human development (Braidotti, 2014; Kurzweil, 2024), as well as considerable doubts about the possibilities of individual ‘Bildung’ (Zuboff, 2018) and the creation of a democratic public sphere (Han, 2021), often place digitalization in an apocalyptic twilight (Manhart, 2023a). Given the diversity of the presumed effects and normative perspectives, it is not surprising that there is still no consensus on what exactly is meant by digitalization (Nassehi, 2019; Wendt and Manhart, 2020; Manhart, 2023a, 2023b).

A more precise version of the term digitalization seems desirable, which is achieved here by clarifying its underlying operability (Chapter 5.2) and embedding it in some of its social and subjective prerequisites (Chapters 2–4). With the aim of such a clarification, the following will take a conceptual-analytical as well as historical-empirical approach. The examples used are all taken from European history (more historical examples can be found in Manhart, 2019b, 2023b). On the one hand, this is an unfortunate limitation of perspective, but further research will certainly remedy this. On the other hand, based on the author’s current state of knowledge, it is precisely some of the changes and events from early modern Europe discussed below that represent central prerequisites for the digitalization of modern society.

In these historical events and depictions, which arose at a time when communication and information processing began to separate, the specific nature of number-based information processing becomes clearer. The spread of calculative practices throughout society, the increasing quantification of the social sphere (Mau, 2017), extending to the scientific self-description of society (Deutschmann, 2025), the global rise of modern forms of organization since the early modern period, and contemporary digitalization are all effects of the increasing reflexivity of the informational form of sense. Information processing has always been effective in the deeper layers of society, but it is now being handled in an increasingly reflexive manner and, at the same time, is imposing its impersonal logic on large parts of society against that of interpersonal communication. Fundamental aspects of the shift from communication to information processing, from facts to data, from *true* nature to *real-possible* virtuality, from face-to-face sociality to digital habitualization, from human to digital intelligence are presented alongside historical examples of their development. The focus here is on the practices of counting, calculating, and measuring, whose gradual differentiation from communication enables the improbable process of the social implementation of an operationally meaningful but at the same time completely meaningless semiosis. Their social and individual cultivation presupposes specific organizational and pedagogical developments that are typical of modern society and lead to the development of modern subjectivity.

The operative separation of Sense and Meaning and thus of information and communication is the central prerequisite for digitalization and thus also for artificial intelligence. This differentiation, which still seemed very artificial at the time of its creation, has been developed, standardized, and socially integrated in the differentiated practices of counting, measuring, and calculating since the end of the Middle Ages. These practices served social functions that were not geared toward digitalization. Rather, their implementation is related to the solution of problems of analogue social forms and individualization techniques, which are exemplary as a form of modern organization and its pedagogy. Objective, metaphorical, and pedagogical measurement (Manhart, 2016) as well as input–output calculations not only enable the structural formalization of modern membership organizations but also shape the cognitive-social habitus of the modern individual. Digitalization is therefore not the continuation of mechanical technology with electronic means. Rather, it requires a complex and therefore highly improbable form of selective connectivity of specific semioses. Sense-making through information processing is isolated from meaningful communication. Sense can be processed without communicating. But this can only be known in retrospect as a prerequisite for development and digitalization. Digitalization is an unintentional structural formation of social evolution, into which the perceptions, desires, and intentions of people in the past are deeply interwoven. Since the late Middle Ages, the number-based form of informatic sense has been systematically cultivated in Europe from language and communication. In calculating with the Indo-Arabic numerals, the meaning-making via signs that is so close to humans was successfully suppressed in the course of the early modern period along with specific practices of measurement. An idea of the complex social and individual prerequisites of this extremely productive differentiation in the sense-generating use of signs can only be developed here using a few examples. The aim is to provide a better historical and systematic understanding of digitalization as an operative synthesis that merges causality and Sense in a sign process.

This makes it possible to contextualize other forms of intelligence historically, including those beyond artificial intelligence. Artificial intelligence currently dominates the discussion about the consequences of digitalization, and so this essay concludes by referring to this form of digital intelligence, because it is primarily in the debate about artificial intelligence that the kind of education and socialization referred to here as digital “Bildung” will emerge in the future. However, intelligence is not understood here as a purely human ability. It is also inherent in animals and, above all, there are intelligent social actors, the most important of which is the modern form of organization. The development of organizations as actors (Coleman, 1979; Luhmann, 2000; Wendt, 2020), the spread of digitalization, and the emergence of social, i.e., in this sense, “artificial” forms of intelligence, are elements of an evolutionary developmental context. This is also the reason why the historical examples cited are mainly from the period of early modern Europe. In order not to overload the text, reference is made to the author’s previous work for details of a theory of organization as an intelligent information processing system (Manhart and Wendt, 2021). This also applies to further explanations of a semiotic theory of artificial intelligence as a system of informational sense-making (Manhart, 2025b). It can be assumed that intelligence is not simply a property of isolated systems but rather the result of a co-evolution in distributed practices. In this sense, the examination of digital actors (AI) leads to a specifically digital intelligence of humans, just as continuous contact with organizations in the past has changed human intelligence, and this also applies in reverse to AI and organizations, as well as for animals such as dogs, which, through constant contact with humans, have developed significantly different intelligent abilities (e.g., understanding pointing gestures) than their wild relatives, wolves.

To better understand the evolution of digitalization, specific cognitive and social effects of the use of signs are described regarding the difference between Sense and Meaning, and the difference between information and communication is profiled by the difference between language and the use of numbers (1). The sense of this differentiation is demonstrated by two prominent celestial practices of measurement (2). The engine of modern number production before digitalization is the synthesis of measurement and calculation, whose semantic productivity is a central element of modern science as an organized process of discovery (3). The operative separation of Sense and Meaning achieved by the monosemiotic concatenation of numerical signs also makes it possible to separate fact and data generation (4). The digitalization based on this means that references nature and the associated truth claims are systematically losing their Meaning in society (5). Data-driven virtual worlds are establishing a new educational milieu that is creating countless new, influential digital actors that will permanently change the way people see themselves: digital ‘Bildung’ (6).

## 2 Productive differences

In the context of AI, it is less about the problems of misunderstood technical simulations (Bender et al., 2021; Deutscher Ethikrat, 2023; Knell, 2024) and more about the possibilities and dangers of operationally independent forms of intelligence (Bostrom, 2013; Metzinger, 2021; Shanahan, 2021; Grace et al., 2024; Kokotajlo et al., 2025; Manhart, 2025b). However, this cannot even come into view if intelligence, thinking, consciousness, and understanding are mixed as mutually presupposing in such a way that only humans can have these abilities. Intelligence without consciousness (Kleiner and Ludwig, 2023; Jin and Rinard, 2023; Manhart and Wendt, 2024), without the ability of self-observation in thinking and the accompanying feeling of understanding, cannot exist for reasons of definition. Human forms of perception, or at least an organically mediated access to the world, are claimed to be necessary for intelligence. It is not self-evident why the variety of technical sensors that can be used by AI should not enable a form of access to the world that is capable of intelligence. It is even less clear why the solution of tasks is not an intelligent solution simply because it is not carried out by a being with an organic body and an associated physical perception (Haase and Hanel, 2023; Manhart, 2023a,b, 2025b; Trinh et al., 2024).

### 2.1 Overdetermination and contingency

The unclear discursive mix has a lot to do with the fact that neither digitalization nor artificial intelligence are clearly defined concepts. In the following, we propose that digitalization should not be understood as the continuation of mechanical technology with electronic means. Digital processes are sense-based semioses as causal relationships (Manhart, 2019a,b, 2023b; Wendt and Manhart, 2020). Sense-generating contingency and causal determination are not contradictory. Digital processes are like organic and cognitive processes, which are no less based on automatic processes and evolutionarily proven stochastics than is the case in digital systems (Manhart and Wendt, 2024). Neither adaptive AI nor humans are mere technology in the sense of causally linked mechanics, but they are both automata (Manhart, 2024). Automata, such as biological cells, are self-acting, i.e., autonomous in their internal processes. This does not exclude causality, but their interaction is complex. Overdetermination is normal and this is precisely where the possibility of contingency lies. Something can be one way or another when it becomes possible to follow several simultaneous effects. Technology, on the other hand, is the attempt to repeatedly allow only certain causal factors to take effect in linear processes. A technology-centric perspective distorts the perception of digitalization, automation, and artificial intelligence toward the side of mechanical causality, while the associated information-processing remains underexposed. Conversely, an operationally free-floating meaning is also an abbreviation. Above all, it serves to remove humans and their abilities from the physical world: metaphysics. Artificial intelligence is a new player in the automaton-rich ecosystem in which modern humans have also evolved over millions of years. Different and sometimes dangerous automata (plants, animals, and organizations) have always been part of this ecosystem (Manhart, 2024).

### 2.2 Sense and meaning

For a better understanding of digitalization, it is necessary to distinguish between two forms of reference of and between signs. Both forms generate sense (Frege, 2007). The first, and in our understanding the most obvious form, is sense as a reference external to the sign: a sign stands for an object or a sign of another kind. The word tree as a linguistic string of signs stands for those objects that are designated in this way. This also applies to the image of a tree, if it refers to something that is not an image. This form of reference always generates a time- and culture-dependent spectrum of reference possibilities, almost all whose individual possibilities are not realized but are co-generating the meaning process. Sense here is an open semantic horizon of sign-dependent possibilities: that is Meaning. Communication, not only in its linguistic form, follows and generates Sense as Meaning. The indeterminacy that is restricted by means of gestures, words, or images requires queries and enables a wide variety of consistent connections with further determinate indeterminacies.

The other form of sense production is based on the strictly regulated concatenation of identical signs, e.g., in calculations with numbers. This monosemiotic sense operates without any reference external to the sign system. Connection and continuation are subject to precise rules and are meaningless, i.e., the continuation is precisely determined: this is information processing. In human consciousness, this continuation only succeeds if meaning making is actively dispensed with or its possibilities are ignored. The difference between Sense as an internal reference (monosemiosis) and Meaning as an external reference (polysemiosis) is inherent in all uses of signs. Words usually refer to things or persons that are not words, pictures to what they represent. They therefore have Meaning above all, although of course words in sentences also refer to previous or subsequent words, that is their Sense. Some words, such as ‘*and*’, ‘*or*’, and ‘*also*’, carry a linguistic function solely in relation to other words. An external reference, a Meaning, is not necessary for this. Nevertheless, they are useful. In everyday perception, the sense of words and images is instantly understood as Meaning, as an open external reference. This is the only reason why it makes sense that abstract art refuses the obvious reference to something outside the artwork, e.g., to similarity. External reference is prevented because it depends on the sense of perception, as the inner sense, the colors and forms of the painting or sculpture. Like some forms of self-referential poems, however, these are artistic peculiarities that do not change the predominantly externally referential meaning-making in verbal and pictorial communication. However, this presumption of meaning does not apply to numbers in modern society. Their monosemiotic sense only became operational relatively late in human history. The social consequences of the differentiated use of the distinction between Meaning and Sense as Communication and Information are enormous.

A social consensus on meanings is difficult to achieve even approximately, and impossible to achieve completely. However, this does not apply to the sense of numerals, which has been reduced to monosemiosis since the early modern period. In “Process and Reality,” Alfred N. Whitehead explains the connection between the easy perceptibility of concrete signs and the lack of certainty in their interpretation: “One difficulty of symbolism,” says Whitehead, “is that the meanings that are difficult to obtain are often vague.” Thus, it is “easier to smell incense than to produce certain religious feelings.” However, “if the two are thus linked,” then “incense is a suitable symbol for such feelings” (Whitehead, 1979, p. 342). It is crucial for the social function of signs that “incense is unambiguous, while religious feelings are not” (Whitehead, 1979, p. 342). The successful link implies the possibility of a reversal of the sign function, which George Herbert Mead, a little later, considers central to learning a socially compatible use of symbols (Mead, 1973, p. 43ff.). Signs then trigger what they only seem to indicate, psychologically they even generate what they stand for in the first place: One smells the incense and feels religiously moved. With numerals, this reversal of the sign function succeeds in the synthesis of measuring and calculating.

Since signs bind attention, they make it possible to relate the perception of different subjects to the form of the sign. The perception of signs is socially significant because it can be accompanied by considerable dissent regarding the individual interpretation and social Meaning of a sign. Many people, for example, smell the incense, see the cross, hear the words and the singing and yet, at the same time, feel and think very different things, without jeopardizing the social event of worship in the least. Modern society makes intensive use of the difference between consensus of perception and dissent of meaning to build structure. As a concatenation of numerals, the informatic sense becomes independently formable and transformable. By strictly linking numbers to numbers, it becomes possible to use their empty formalism to build social structures. Meaninglessness becomes socially significant. The referencelessness of numbers makes it possible to continue working with the strictly coupled information alone and thus to stabilize highly formal cognitive, pure mathematics, such as social structures, e.g., organizations. For the deep layers of social structure formation, communication becomes increasingly inconsequential. This allows communication to be freed from social considerations, i.e., the growth of its diversity in the modern age. In digitalization, it is the repeated application of monosemiotic Sense to itself that now allows digital realities to proliferate alongside the worlds of mathematics.

### 2.3 Information and communication

In all traditional cultures, the presumption of meaning toward signs is predominantly specified as an expectation of communication and generalized to everything perceptible. The perception of something as a sign goes hand in hand with the assumption that this sign is associated with an intention that needs to be understood as a communication. Sense is then the sense of communication, the understanding of meaning, and the development of intentions. Everything communicates. Mountains, springs, trees, and animals are like humans in this respect: they have spirit and intentionality. Intentions must be recognized by observing and interpreting signs. The form of sense in society that guides cognition and creates structure is language, from which image (hieroglyphics etc.) is derived into the form of writing. Everything that exists has Meaning and thus a part in communication. Numbers only play a role, if at all, as carriers of meaning. The mysticism of numbers that was widespread in the late Middle Ages and early modern period (Bruno, 1991; von Kues, 2002) understands numbers as indicators of meanings that work through them, which in turn require verbalization to be understood. This does not exclude very practical uses of numbers and counting processes (Wedell, 2011), but society sees itself as an order of communicative actors and therefore everything still belongs to it and communicates. Self-descriptions of these societies therefore only know levels of relevance of the expressed sense but not a communication-free, unintentional environment. The world is cosmos and, as such, communication, society, and intentional creation are one.

In fact, however, it is precisely the formal increase in the presumption of intentionality in the mythologies of the high religions that provides the first signs of a resolution. A rift runs through the cosmos of meaning when the gods flee into transcendence, as is often the case in creation religions. By relating the ubiquitous presumption of intentionality to beings hidden behind the perceptible (spirits, gods, the one God) an initial distancing from the exuberant sense of communication becomes possible. A burning thorn bush can then become a sign that someone, but not the thorn bush itself, is communicating something through it. If one observes semiosis with a view to the unity of the difference between consensus of perception and dissent of meaning, one can see that modern society is growing in a sign-specific expansion of this difference. Certain signs appear less and less as communication, i.e., the presumption of intentionality is gradually inhibited. In modernity, there are then signs that stand as signs for not being signs for something other than certain other signs: These are numbers. In their current version, numbers only refer to numbers. External reference must be indicated separately with other types of signs, e.g., letters for units such as m, kg, or kw. In the modern age, the generation of meaning no longer works with numbers alone but requires communication.

Originally, however, ‘numbers’ were indicators for things that are counted with the help of other things, e.g., with fingers, stones, or lines. Counting requires the perception of the simultaneity of different things. As numbers cannot be understood without Meaning, i.e., what is counted, the number signs vary depending on the object being counted. Communication and information are not separated at the level of signs; everything appears as communicable Meaning. Freed from any Meaning, a number is a difference that has only this subject: a three is not a four and not a two. When reduced to the sense of information, no communicative sense and no further intention is expected when three and three make a six. As numbers, numerals therefore mean absolutely nothing. Their sense only refers to other numbers. This is precisely why, in principle, everything can be denoted by numbers, i.e., counting: 3 × 3 is and always will be 9. Whatever an observer associates with the numbers, i.e., whatever Meaning they give them, the connection logic of the numbers, their reference to other numbers, is not affected by this. In language, on the other hand, the exact sequence of signs, sounds, and words is only of minor importance for a socially and individually connectable interpretation. Words and sentences that are phonetically, grammatically, or syntactically ‘wrong’ are also understood. Language and parole differ (Saussure, 2013). Depending on the meaning, i.e., external reference, the word and language structure also changes and, depending on the language, meanings that are difficult to reproduce in another language are also possible. For the informatic sense of numbers and calculations, on the other hand, the exact arrangement of the digits and the correct use of the linking signs are crucial. Otherwise, the result of a calculation is incorrect. Numbers in calculations only ever generate and transform their own sense. That is why the information content of a calculation is the same anywhere else in the world. In addition to the reduction of meaning and inhibition of communication, the necessary concentration on the precise sequence of characters makes the individual learning of the informatic form of sense more difficult. The memory of many endless hours of mathematics lessons in which the question of why remained unanswered may suffice as evidence of this—for most people.

With the semiotic separation of communication and information, of Meaning external to the sign and Sense internal to the sign, both forms can now be increased independently of each other in their capacity for resolution and recombination. The detachment of numbers from the external reference creates the possibility of expanding the sign system exclusively with the means of this sign system: Numbers are ordered into ascending series along their different connection logics; different series are systematically related to each other (natural numbers, rational numbers, real numbers, etc.) and these connected series are in turn counted as new series, and so on. But what was the purpose of this expansion of mathematical possibilities? Whether the infinite variety of these forms and worlds of pure mathematics corresponds to something outside them, whether this or that quantitative connection is an icon of something in the world outside mathematical signs, cannot be decided by mathematical means alone (Peirce, 1986, pp. 240–246). The expansion of the combination possibilities of numbers has therefore long been tied to the solution of practical problems, to simplifications of the handling of large quantities of real things, to religiously motivated problems of celestial geometry or architecture, for example.

## 3 Social functionality: attention control through signs and practices^1^

### 3.1 The productivity of cultural appropriation: the non-linguistic notation of numbers

The introduction of Arabic-Indian numerals in Europe was accompanied by the gradual development of number-specific linking signs (+, −, x,: etc.), which can be understood as an indicator that the use of numbers was being detached from the linguistic-communicative enclosure. The new notation makes it easier to concentrate on the number-specific, informational sense of connection. However, the protracted history of the implementation of a language-independent mathematical notation shows how difficult it is to separate communication and information processing from one another. Modern mathematical notation has been *explicitly* established *against* linguistic notation since the 15th century. “The period between 1,460 and 1,550 is called (…) ‘German Coß’, because the experts active at that time (…) succeeded in making the Austrian, southern, and central German language area the center of a development that set itself the goal of detaching mathematical terminology from the written word” (Kaunzner, 1992, p. 159f.; Wußing, 2009, p. 331ff.). The beginnings of this development can be found in the pedagogical arithmetic books of Heinrich Petzensteiner and Adam Riese (Petzensteiner, 1989; Riese, 1522, p. 32ff.).

The Indo-Arabic way of writing numerals, which is not common in Europe, is unencumbered by the linguistic and cultural traditions of Roman letter arithmetic, so that the Meaning of the number word and the number sense in the logogram of the numeral can be consistently separated from each other. By using number-specific linking signs, written numerical arithmetic becomes independent of any language. The liberation of numerals from any Meaning promotes their use in the stubborn transformation processes of extensive paper calculations up to mechanization as a calculating machine. As a highly precise form of writing, it replaces the linguistically vague, communication-oriented formulation of calculations. However, it is not the cause of this development. There can be no question of a widespread and rapid introduction of arithmetic with Arabic numerals in the late medieval economy (Arlinghaus, 2002). But the Meaning of economic interests and needs in the introduction of numerical arithmetic should not be overestimated, as the long-lasting practice of line arithmetic with Latin letters in this area shows (Hess, 1977).

### 3.2 The measure of the soul: if gods measure, they don’t count

This can be demonstrated by a religious tradition of measuring, in which its communication-inhibiting effect, the speechless production of consensus and substitution of decision-making appears exemplary insofar as it also applies to gods. In the Iliad, Homer reports on an Olympic conflict that breaks out in connection with Hector’s battle against Achilles. Two camps form in the heaven of the gods: those who want to give victory to Hector and those who want to give it to Achilles. Zeus, on the other hand, cannot make up his mind, so that the struggle between the two heroes, who are circling Troy, and between the gods themselves, threatens to go on indefinitely. Zeus then resorts to a surprising technical device: “But when they [Hector and Achilles, S.M.] came to the springs for the fourth time, Zeus the father held out his golden scales to them all [i.e. the assembled gods, S.M.]: in one bowl he placed the fate of Achilles—in the other bowl that of Hector, the tamer of horses. He held them in the middle: and the bowl in which Hector’s fate lay sank down to Hades. Apollo now forsook him…” (Homer, 2008, XXII, 208–14, p. 455). This ends all discussion; the perception of a measurement decides, not Zeus or any other god.

A functionalization of a scale as a measuring instrument, which has a different religious basis but is almost identical in terms of creating a speechless consensus of decision, can be seen in the depictions of the Last Judgment on the west walls of numerous French cathedrals but also on and in other Catholic and Orthodox churches. Possibly inspired by Egyptian images (Link, 1997, p. 133ff.; Kretzenbacher, 1958, p. 150ff.), a scene in the Last Judgment can be found, first on a tympanum in Autun Cathedral, which seemed so impressive to contemporaries that it became tradition-forming for centuries. This is all the more remarkable as there is no surviving textual, biblical, apocryphal, or highly theological model for this pictorial representation. In the stone carving, created by the sculptor Gislebertus around 1,130 and probably designed by the local bishop Etienne de Bage, Jesus is seated in a mandorla in the middle of the tympanum. It is the Day of Judgment, and the Lord God is judging the living and the dead. He sends some to paradise, which is indicated to his right, and others to hell, which is to his left. As if to reaffirm his role as the lone decision-maker, the mandorla surrounding Jesus is framed by a banner that reads: “I alone order all things. I crown the deserving. I as judge bend the criminals under punishment.” However, what happens for the first time in such a depiction to the left below immediately casts doubt on this. There, the archangel Michael and the devil are standing opposite each other, both busy observing a large pair of scales between them or, in the case of the devil, filling them with souls and manipulating them. In the two scales are figures that probably represent the embodiment of the soul or, more precisely, its deeds. What happens is clear: if the scales tilt to Michael’s side, i.e., if they sink to the right, the soul can enter paradise, but if they tilt to the left, the devil, who therefore wants to pull the scales downwards, can take the soul to hell. However, it is completely unclear what theological function this process can have. Does the Last Judgment consist of two judgments? What purpose could the weighing of souls serve if God knows everything and, as written, “alone orders” and bends the “criminals under punishment”? If we turn the situation around, the theological problem becomes clearer: if the scales tip under the weight of good and bad deeds, what does God decide? Is his knowledge and judgment not then superfluous? About people’s ability to judge and make decisions, the same problem naturally arises when using measuring instruments.

### 3.3 The devil’s share: measurement as substitution of judgment

It could be argued that the distinction between temporal and eternal punishments for sin that emerged from the 10th century onwards (Angenendt, 1997, p. 652) could also justify a twofold judgment in the Last Judgment. The remission of merely temporal punishments for sin through the granting of the *thesaurus ecclesiae* (ibid., pp. 652–657), which, incidentally, can be counted and divided into pieces, in the ecclesiastical indulgence, presupposes God’s judgment on the remission of eternal punishments for sin. But there is no contemporary evidence for such an interpretation, no theological discussion of the pictorial program of interest here have survived. The theologians remained speechless in the face of what is shown there. For in the sequence of the rich tradition of medieval depictions of this scene, one can observe how the measuring, i.e., the weighing of souls, increasingly pushes the divine judgment or, more precisely, the figure of God, into the background. On the tympanum of the Cathedral of St. Etienne in Bourges (ca. 1250), to name just one prominent example of many, Michael with the scales is in the exact center, while above him, but still on the same axis, God is enthroned. The arrangement seems to be similar in the later large paintings of the Last Judgment, e.g., in Rogier van der Weyden (Beune, ca. 1450) or then in Hans Memling (Danzig ca. 1470). There, however, the archangel with his scales is not only in the center; he also moves the scales and the process of measuring into the foreground, clearly enlarged, objectively and spatially. Although God is still visible, he already seems to be disappearing into heavenly spheres. And so it is only logical that, alongside and after this, we increasingly find depictions of the archangel with his scales in which God is completely absent (e.g., the main portal of Bern Cathedral by Erhart Küng 1460/81 or the Archangel Michael by Holbein the Younger ca. 1523).

The perception of measurement not only attracts attention and collectively renders gods and humans speechless; it also replaces divine and human judgment with the process of measurement. Individual and collective decision-making is replaced by the measuring of differences. Even the one, all-powerful God loses relevance through the practice of measurement. Even at the Last Judgment, we no longer look at people’s concrete lives, we no longer tell life stories, we no longer eloquently compare individual sins and good deeds. With the use of a measuring instrument, however, there is a tendency to follow its signs alone. This is what happens in the Egyptian realm of the dead, in the Greek heaven of the gods, and in the case of the Christian Last Judgment. The technical unambiguity of the measuring apparatus replaces the inconclusive judgment based on interpersonal communication, which is always vague in its interpretative references, i.e., open to dispute. Measuring creates a perceptible difference between before and after, between conflict-prone vague communication and digital unambiguity. The communication-inhibiting and at the same time consensus-building effect depends on the technical unambiguity of the difference created in the measurement. This difference is experienced as an evident imprint of a factual connection, the clarity of which no longer requires discussion.

Encoding this difference in Arabic numerals not only increases the symbolic unambiguity but also allows the interpretation of the results to be replaced by calculating back to new semantic entities. This is because the religious measurement practice considered here is also a metaphorical measurement (Manhart, 2016, 2023b). The weight of a soul is not understood here as the weight of a soul but as a measure of a good or bad life. Weight and good deeds have nothing to do with each other but are understood as an indexical connection in the interpretation of the measurement results. Physical heaviness is seen as an effect, the falling of the scales is then a sign of degrees of morality or the fulfillment of a life pleasing to God. The fact that this index does not exist for today’s observers is related to the verifiability of such assumptions of effects by means of statistical procedures in the back-calculation of measurement results. This requires the synthesis of measurement and calculation, which it is possible to regulate the practice of metaphorical semantization described here, i.e., the inference of measurement results with an affinity for effect to causes by means of calculation rules. The fact of the measured result is the basis of the perceptual consensus on which the structures of modern society stand. However, the communicative interpretation of the measurement, the consequences of its metaphorical recalculation to causes—but not its numerical result—can then be argued about. It is no coincidence that the Devil is using the measuring device alongside the archangel Michael.

## 4 The productive synthesis of measurement and calculation

The synthesis of measuring and calculating enables the development of new things through regulated metaphorization (Manhart, 2016, 2019a, 2023b). The open space of meaning of communicative metaphor, which previously allowed, for example, physical gravity and morality to be brought together by measuring with a scale, is organized along the numerical grid and extremely reduced by the strict formalism of a calculation. If the results of measurements are calculated, they can be compared more precisely with the results of other measurements. This increases the relevance of further measurements to confirm or correct their results. Measurement therefore promotes calculation, which in turn promotes measurement. The merging of measurement and calculation does not solve the problems of language-based logic that have been discussed since antiquity, but it does circumvent them through productive practice. In his dialogical syllogistic, Aristotle was already concerned with finding a way to infer yet unknown premises from known facts or from a final proposition (Kapp, 1965, p. 84f., p. 87). Inferring a *true* conclusion from given premises is the usual form of deductive proof. Only this direction of inference can be controlled by regulating the language. However, something new can only be inferred by reversing the deductive scheme. Inferring something invisible from visible signs is the procedure to which metaphorical measurement owes its semantic productivity (Manhart, 2016). In this respect, metaphorical measurement is a numerically regulated abduction procedure.

The unknown, invisible entity becomes recognizable by its effect. Measurement is understood as the recording of this effect (Manhart, 2019a). The measurement or the measuring device records an index that is fixed as a numerical value and can be reused. The vague linguistic assumption of a cause can now be supplemented and finally replaced by a calculation. The cause is stabilized procedurally by repeating measurement and calculation as uniformly as possible, specified via further effect measurements and finally designated and communicable as a uniform entity. The procedural repeatability of measurement, like any calculation, serves to objectify, i.e., to largely exclude subjective but also social factors, such as language-guided communication. The diverse individual perceptions of effects become calculated constructs, which are now regarded as the causal factors behind the numerical measurement results. The modern concepts of energy, mass, attraction, or power are measured entities which, as overarching, abstract constructs, elude all perception. The share of perception and consciousness in the meaningful development of a new world is already reduced to a minimum here. In contemporary science, which is far removed from perception, it is reflexive calculation methods, i.e., indexical criteria such as retest reliability, that serve to ensure social acceptance of the assertion of the existence of ever new and nonsensical entities. The expansion of this practice in the rise of the natural and social sciences, however, originally depended on the social consensus generated by measuring and calculating in the perception of measured numbers. Above this consensus, the synthesis of measurement and calculation becomes an extremely productive process of discovery.

### 4.1 William Harvey (1628): the synthesis of measurement and calculation as a method of discovery

In 1628, William Harvey’s “Discovery of the Circulation of the Blood” is an example of the argumentative power and scope of a strict coupling of measurement results in arithmetic. If one follows the connection rules of informatic sense, the imperceptible can be precisely *inferred*, i.e., unambiguously *designated*. Harvey had no knowledge of capillaries, as he did not yet have a microscope at his disposal. Blood circulation cannot be seen with the naked eye during autopsies. He calculated the circulation, i.e., he deduced it as a plausible way of explaining the numerical results of his measurements and calculations. Harvey explicitly emphasizes the difference to the always vague derivations on the basis of linguistic descriptions: “But lest anyone should say that we offer only words and make only delicious assertions without any justification, and that we introduce innovations without good reason,” he wanted to make his ideas “obvious” by means of a calculation: “Let us suppose (whether in thought or experimentally) that the quantity of blood which the left ventricle holds in its dilated state (as soon as it is full) is 2 or 3 or 5 ounces. I have found over 2 ounces of it in the dead” (Harvey, 1910, p. 56). Based on his own measurements of the amount of blood pumped by the heart with each beat, he calculated the total amount of blood in the body. This amount of blood must circulate, as neither its continuous production nor its consumption in the body can be plausibly explained. His “discovery,” which is a calculation, is accepted in parts of the scientific community even before blood circulation can be seen under a microscope.

### 4.2 Gottfried Kirch (1672): the ease of transition from measuring to calculating

The description of a measurement that appeared a few decades later, in 1672, in Gottfried Kirch’s ‘Christen-, Juden-, und Türken-Kalender’ (Calendar of Christians, Jews, and Turks) serves to further disseminate the cognitive and practical skills for the practical application of the informatic sense of measurement. There he reports in detail on the observation of a “lunar eclipse that occurred early on September 19, 1670,” the “beginning/middle/end and currency of which he was able to observe precisely without any astronomical instrument, merely by means of a “common striking clock,” which he wanted to present to the interested readers of his calendar for imitation. Gottfried Kirch, who was later to become the Royal Astronomer of the Brandenburg Society of Sciences in Berlin, describes the course of his measurement of a lunar eclipse in a writing calendar, a small, regularly published publication for an educated public (Herbst, 2010, vol. 2, p. 171f.). The presentation of the measurement is pedagogical, it is intended to be understood by the readers, which is why he does not use a telescope, which his readers do not possess. The example shows how much needs to be learned for a pragmatic understanding of measurement. The cognitive complexity of the measuring process quickly becomes clear, even though no complicated instruments or intentions associated with measuring are used. Kirch observes with his naked eyes and uses a wheel clock to measure time, as many citizens had already set these up in their homes. Both Harvey and Kirch made use of another ‘symbolic machine’ (Krämer, 1988) that was accessible to everyone, even if it was not yet widespread. The routinized use of this drawing machine, the mental use of the number line as a universal scale or as a paper computus, fundamentally changed the subjective and social role of measuring.

Kirch’s wheel clock, which is probably not yet a pendulum clock, presumably only shows the hours, as was customary at the time. Minutes, seconds, and the smaller time divisions required for astronomical calculations had to be counted and calculated using the sounds of the clock. His description of the measurement therefore flows smoothly into a calculation that still makes use of the linguistic linking of numbers, combining Arabic numerals with connecting words: “The rising wheel in this clock has 35 ticks, double that number gives 70 ticks in one revolution, which multiplied by the 100 h = wheel ticks give 7,000. These are divided by the sixth drive of the escapement wheel and result in 1,166 2/3 ticks of the balance in 1 h, which is exactly 19 4/9 ticks per minute, where one tick corresponds exactly to 3 s 5 1/7 thirds or 3 s 5 thirds, 8 fourths, 34 fifths, 17 sixths, etc.” (Herbst, 2010, p. 171). The accuracy of his specification of the duration of the eclipse as “2 h 47 min 32 s 2 tert.” cannot be measured with his watch but is due to the synthesis of perception, mechanical registration, and semiotic calculation. The aim is a clear result that has been purified of its own subjectivity and freed from the limitations of perception: a number. Those who repeatedly abandon themselves to the logics of mechanical and semiotic machines by observing their movements and following them cognitively by counting and calculating in writing learn to choose the conditions of their own perceptions in such a way that they are no longer just their own, i.e., that they coincide with the equally regulated observations of others. In measuring and counting, the subject becomes its own object.

### 4.3 The calculated time of modernity: what happens when measuring with numbers?

Only if one steps back from one’s presuppositions can a modern observer, long since habitualized by countless measurements, recognize how presuppositional, i.e., in need of getting used to and learning, it is what happens and must be thought of in this and in basically also in every other measurement. The comparison of two movements, the movement of a clock and the movement of the moon through the earth’s shadow, can only show that there is no similarity between these two movements. What does the turning of several cogwheels have in common with the movement of the moon, so that we can learn something about the other from one? The sound of several gears turning inside each other is related to a noiseless, visible movement in the sky, a distant process in space. The sequence of “tickings,” i.e., the sounds of the gears, is used to standardize a complex connection of several individual movements, the simultaneous rotations of the gears, into a sequential form or, more precisely, to break this movement down into equal, repeating units that can then be counted in sequence. Only the orderly process that emerges from counting serves as a yardstick against which the continuous movement of the moon can now also be observed, broken down, and transformed into numbers. The use of this symbolic third party takes on a new Meaning when the counted sequence is thought of as a third movement: *time*. This empty and thus all-containing continuous time is ordered and sequentialized as a series of numbers. It can be subdivided more and more precisely, i.e., in smaller steps, if the abstract-continuous time space and the numerical space, which can in principle be divided infinitely further, are identified with each other in practical measurement/calculation. In measurement, observation, which begins with visual perception, is increasingly abandoned to calculation, which is distant from perception. The world appears as a record on paper.

The infinite divisibility of the continuum of the number series works for the modern relativization and technical-semiotic narrowing of perception for cognition. Under the conditions of enforced pedagogical measurement, the pervasive control and management of the movements and work steps of personnel in organizations (schools, sports clubs, companies, etc.) by means of long-running measurement regimes, this also increasingly affects self-knowledge, the perception and assessment of the subject in and of itself. Even Gottfried Kirch’s divisions of time below tertia no longer correspond to moments of perception, i.e., experience. This detachment of the sense of measurement resulting from sensory perception is supported by the persuasive power of externalized measurements and the tendency to substitute judgment. With the digital feedback systems available in every area of everyday life in the present, every further individual change is monitored on a screen and oriented *instantaneously*. Because we now not only seek precise knowledge but can also semiotically represent it in novel ways, the resulting insight distances itself from prior notions and experiences, leading us to increasingly distrust them. This is because the number series can be handled mathematically without any problems, right down to infinite detail. Even the digital self-measuring devices that are close to the body only produce strings of characters on screens that people follow. You perceive the number signs, tables, and progression curves and correct your Self according to this image, at any time and again. In doing so, you take note of changes with a precision that is not without consequences for the acceptance of the inherent vagueness of cognitive-emotional self-perceptions and attributions. Measuring and calculating shifts the source of meaning from the observation of the interior to external signs of semiotic machines.

This measured and calculated sense is extremely productive, both subjectively and socially, precisely because of its simple-minded precision. The sense generated in the informatic form is precise but also simple-minded and unsatisfactory in comparison to the sensual fullness of subjective perception. This creates a considerable need for communication and drives literature and art production. The consensus on the simple-minded sense of the signs also offers the possibility of further cultivating diverse differences in the individual interpretation of numerical measurement results. Individuality can be further increased and communicated in the production of meaning without jeopardizing the social consensus on its numerical sense, i.e., the processing of information. The exuberant communicative-vague production of meaning therefore always generates a further need for more measurements, more low-reference sense.

## 5 Facta, ficta, data, fake

With the systematic use of number-based forms of measurement since the early modern period, the emergence of number-based registries and accounting methods in the modern organizations that are now spreading everywhere, and the infiltration of these informatic practices into almost all areas of life, the growth of a number-based network of social information exchange and structure building is accelerating. The perceptual consensus that clings to concrete numbers and measurement practices also enables the cultivation of divergent interpretative discourses. Communication is detached from the considerations of this consensus: the path to enlightenment, freedom of opinion, and artistic freedom. The prerequisite for this is the differentiation of an increasingly automated, i.e., decision- and discourse-independent, informatic structural update of society from a semantics that is increasingly driven up for this very reason. The new informatic semiosis changes the forms and contents of self-perception and self-description of individuals and society. Subjectivity and sociality are reformatted by readjusting the relationship between factuality and validity by means of a flood of measured and calculated data.

### 5.1 Maria-Theresia (1745): from telling to counting

“Nothing has happened in Italy either. I want to know where all the money […] is or whether it has arrived. I want to see the data.” (Stollberg-Rillinger, 2017, p. 119). Maria Theresia wrote this in a letter to Dietrichstein, the President of the Court Chamber, in the middle of 1745. For her, data seems to be what it is for us: Numbers. However, in the early days of the use of words in the 17th century, *data* were not numbers but linguistic sentences that served as the starting point for investigations (Rosenberg, 2014). The dogmas of religious argumentation are referred to as *data* because, ideally, they are given by God. They are an unquestionable starting point, the material for further reflection. Regarding the latter understanding, it is perhaps no coincidence that the earliest *current* use of the word “data” in the sense of “a heap” can be found in a theological work from 1,646 (Rosenberg, 2014 p. 136f.). In the 17th century, the word still appears in parallel in linguistic and mathematical writings and is thus still understood in both forms of sense as communication (sentences) and as information processing (numbers). Today, however, data are practically exclusively numbers whose function is to be available as a ‘pile’ of material (big data) for further relations, i.e., calculations.

### 5.2 Untrue facts are true fictions

As products of a measurement, numbers are facts. They are clearly made (*facere*). At the same time, they can become data. Data is perceived as given (from *dare*), as reality in the literal sense. Their being made recedes into the background. As early as the emergence of the term in the 17th century, *data was* understood as a loosely linked set of elements, as material to be worked with. The understanding of big data as an unstructured ‘large quantity’, which is only made usable through selective linking, is its modern version. But why is it that strictly linked numbers are seen as loosely linked data? How and why were these seemingly contradictory concepts brought together as a sense of information? And what is the purpose of *data* if *there is* a dual of *facta* and *ficta*?

First, designations are always facta, i.e., made, and can therefore also be ficta, i.e., referentially empty. About the possibility of fictionalization, it is again instructive to distinguish between communicative and informatic forms of sense. Fictionalization is empty referentiality. Referencing does not make physical contact with an external world but represents the possibility of designating something as outside the functioning string, that is sense, or as outside the functioning form of sense: This generates Meaning. An empty reference is a sub-form of meaning-making in which there is no concretization of the signified outside the meaningful sequence of signs, i.e., as a fact, e.g., through demonstration or measurement. In the case of empty reference, possibility and reality are thus systematically drawn apart without separating them completely. This difference is easily created in language. With the distinction between yes and no, which is specific to language, it is permanently on the horizon of every verbal communication (Luhmann, 1997, p. 205ff., esp. 221ff.). However, this does not mean that a distinction is only made verbal between real and possible; rather, this difference is present in all communication. Numbers, on the other hand, do not allow fictionalization, precisely because real and possible make no difference in calculations, i.e., they are simply the same thing. The Sense of numbers is always pure self-reference. Numerical sense knows no external and therefore no empty reference (Meaning). There are therefore no mathematical fictions. Concrete calculations and functions are not dependent on any external reference in their logic of connection, their sense. Sense and Meaning do not interfere mathematically, i.e., Meaning makes no mathematical difference. The Sense of the difference between Sense and Meaning is not empty or indeterminate informatically; it is none.

### 5.3 True data does not have to be true

The fact that there is no difference between real and possible in the informatic form of sense reinforces the tendency already described in dealing with measurements to perceive their signs not as possible but as real. Numbers appear in perception as given, which promotes the synthesis of data and numbers. In the modern age, both digital fictions (images, films, games) and the worlds of science consist of numerical data. This is possible because data is neutral with regard to the distinction between true and untrue. Even untrue or false data remains data that can be used to create fictional worlds or fake news, for example. As data, numbers are merely material. This is countered by the fact that the informatic form of sense, due to its lack of external referentiality, knows no difference between truth and correctness. Facts, on the other hand, are sensitive to precisely this difference. Something is not a fact if the external reference of the intended sense, its Meaning, is not true or is empty. False facts are not facts, while empty facts are fictions. In verbal terms, facts are therefore always sentences with the Meaning of being true. Being true is a Meaning that can only be assigned to numbers as facts. To achieve this, they must become part of the communicative form of sense, e.g., by being linked to units. Mathematically, correctness and truth are identical, linguistically they are not. There are countless linguistically correct cases that are factually incorrect. Informatically, this makes no sense. If it is rule-fulfilling, i.e., correct, then it is also mathematically true (Wengenroth, 2006, p. 256).

Through fusion with numbers, data becomes a reference-free and therefore truth-related neutral material of meaning. In this respect, data is the elementary form of the modern world. Data is the modern substitute for nature. It takes the place that was called substance or *materia* in ancient and medieval philosophy. Pure substance, like modern data, takes up the relations of all the forms that make up the world. Insofar as data are numbers, the world related from them is dependent on the possibilities of linking the numbers. As given elements, data are real. Data are *naturally* made signs. In contrast to facts, however, whether true or false, data remain data. They are what they are. Nature cannot be wrong either. It too is what and how it is. Nature cannot be untrue; it can only be misrepresented. Failure of perfectibility or corruption are the morally connoted problem formulations of tradition. However, this presupposes a different view of this nature, namely, to see it as creation and thus as a made fact. On the other hand, nature is a datum. It is precisely this distinctive neutral being that is meant when reference is made to nature by referring to numbers as data.

The conversion of data into facts and back into data is the central process in the creation of scientific truth. The nature of science is a cloud of data from which truth is calculated. When data becomes true, it is a fact, i.e., it is understood as a created relationship and can therefore be true or false. Modern natural sciences, or more precisely information sciences, are therefore ideally pure data sciences. Their results are predominantly expressed in numbers. Numbers are generated by measurements, and conversely, data is expected to be the result of measurements. The separation of scientific-informational and communication-centered worldviews arises from the standardized comparison of numbers. The practice of measurement replaces vague pictorial and verbal analogies in the justification of world descriptions. Without the establishment of an informational representation of the world as a causal network in which numerical data is calculated, it would have been impossible to isolate science from the magical worldview that assumed communication and intentionality everywhere. Numerical signs are treated as non-signs because, as measurement results, they are indices, products, and indicators of effects: they are given. As data, the conscious production of numbers in measurement processes is not usually questioned. This also applies to the unprovable claim that the possible links between numerical data correspond to the relationships between the “real objects” they index. The implicit assertion of the correspondence of every statistical data analysis fits in with this if its results are communicated as facts. Regarding the correctness of the calculation, the fulfillment of ambitious coherence requirements, alongside classical correspondence, constitutes a socially consequential mixture of informational correctness and communicative truth criteria.

## 6 Neither fact nor fiction. Digitalization, data worlds, and their social consequences

### 6.1 Another productive difference: more data, more communication

Society deals with self-generated data; it deals with itself. This has always been the case, but now it knows about it. The reactions to this utilize the full range of what is semiotically possible. Data binds attention as numbers, creating a communication-inhibiting perception that enables social consensus and individual deviation at the same time. Since data can be facta, ficta, or fake, meaning deficiency and meaning surplus, strict concatenation and vague meaning production, generality and specificity, sociality and individuality can all be increased simultaneously in modern society. But the differentiation of sign-based Sense has its price. The self-description of society as a communicative system and its environment as a countable data network not only means that the gods and spirits disappear from nature, it also means they are migrating into society and its subjects in the form of growing claims to meaning. There they now weigh down on all their actions. The containment of the reference surplus of communicative meaning production leads to an erosion of the meaningfulness of world perception as well as to the loss of the worldliness of the informatic sense—a double emptying of sense that throws back onto society all those expectations of sense that bounce off the strict (weak) sense of the informatic form. Imagining the world as a strictly ordered layering, superimposition and relation of number sequences does not have much that is sublime about it for most people and offers no room for a subject-related sense of communication for the understanding of Meaning. People are therefore happy to leave the transformation of these sign configurations to semiotic machines. The endemic problem of meaning in modernity, the individual feeling of meaninglessness in the abundance of communicated meanings, is rooted in the cultivation of the informatic form of sense. As a digital network, it is growing rapidly. The problem of meaning is a problem of communication. Nobody talks in numbers or digital data. The informatic structure lacks the Meaning that one could indulge in understanding. AI can change this at the cost of further relativizing the human subject and ‘its nature’ (Manhart, 2023a; Manhart and Wendt, 2024).

The loose coupling of the psyche to the data perception consensus allows individuals to indulge in the production of meaning through communication. Especially in times of number-based digitalization, the quantity and artificiality of meaningful communicative forms is therefore increasing. Verbal and visual representations are increasingly cultivated, precisely because their relevance for the structural development of society is diminishing. This applies to the growing production of literature, the media creation of public opinion on countless mass media channels, ongoing communication on social media, and the steady increase in contributions from the social sciences and humanities. Communication always takes place everywhere, which is why and precisely because the relevance of every statement for society is dwindling. At the same time, pedagogical measurement provides the appropriate data-driven practice of informatic self-assurance (Manhart, 2016; Manhart, 2023b; Schäffer, 2015) as a synthesis of technical external and participatory self-generation, of which the quantified-self-movement is just one example. The pedagogical measurement, which is aimed at changing the object, corresponds to the fact that fixed-coupling numbers are read as loosely coupling data. Feedback data is available for change in the next measurement, whereby certain quantitative changes are interpreted as improvements in internal psychological qualities. The fact that these possibilities for change are based on only small differences coincides with an idea of individuality that digitally registers even the smallest deviations, even the smallest random microdiversity, and thus makes them interpretable as identity-relevant learning or potential for change. Organized external control by means of pedagogical measurement regimes and the subjective need for external sources of self-assurance go hand in hand. Participatory data production is experienced as self-efficacy. The comparison of past and the expectation of future results generates individuality as a streamlined trajectory in the data cloud.

### 6.2 Semiosis and causality: the digital process

The blind spot of such subjectivation and communication-centered self-descriptions of society lies in its material, i.e., that which appears to be given without residue: data. This data is now primarily generated digitally: a materialized, electronically ordered stream of signs, zeros and ones, arranged by switching electrons (Manhart, 2025b). Virtual is material, i.e., the difference disappears. Therein lies the specific point of digitization. The special quality of digitization lies in the synthesis of the strict sign logic of numbers (the concatenation of zeros and ones) with the material-inherent causality of electrons. The material substance, the electronic connection, is no longer merely, as in the case of paper, the carrier of information, it *is* the information. The electronic-digital network is a senseful causal dynamic: what is digitally informed, i.e., organized as a sequence of electrons, is causally interlinked; it changes instantaneously and automatically as soon as an input is made. Character sequences can only be changed within the limitations of electronically materializing semiotic programs and the processors that process them, while data input at other locations has an instantaneous influence on a multitude of other data. The semiotic network is a material network and its extension is digitization. In its most advanced forms—as in the case of self-learning AI—this hard software network organizes, i.e., programs, itself (Wendt and Manhart, 2020).

The social consensus, laboriously established as modernity, on formulating the difference between system and environment, between self-reference and external reference by means of the differentiation of communication and information as a contrast between nature and society, is also undermined by the understanding of numbers as data. Digitally, nature is only established in society in numerical form, i.e., calculated. Social movements and many educational programs react to this by remythologizing or normative charging of humans and nature. Nature is morally reshaped to be able to mark an effective difference at all. However, this is the fading stage of an originally obvious distinction and is politically extremely risky because factual differences are transformed into conflicts of values. ‘Nature’, however, is endangered because the semiotic-number-based technology of the digital production of virtual worlds marginalizes the difference between society and nature at the level of sense. Because this difference makes no difference, it no longer has any informational value. The digital semiosis is as material so natural as it is virtual. It is therefore not a question of returning to free, even untouched nature, i.e., implementing educational programs that primarily reduce the time that adolescents spend in ‘merely’ virtual, digital worlds. This will not work without doing without the analogue ‘rest’ of society, which also includes the individualized subject.

This development reinforces the introduction of AI. The debate about the possibility *of artificial* intelligence, even of artificial consciousness, lives beyond operational and factual arguments, above all from the normative charge of the difference between nature and technology. In this, mankind itself becomes a myth (Manhart, 2023a, 2024), while the apocalypse of the digital singularity is eagerly awaited. This expectation is part of an exuberant crisis discourse (Manhart, 2023c), whose charged rhetoric and normativity is constantly increasing with the relativization of the difference between truth and fake in digitalization. This is primarily because the fact that the data-driven digital society is constantly confronted with the contingency of its own distinction between self-reference and external reference. This is because the traditional concept of truth also hinges on this difference. In the present debates about fake news and the status of scientific findings, it is not just a question of what is true. The main issue is whether the difference between the sequence of signs and ‘external nature’ that is claimed for a correspondence theory of truth, whether the Meaning generated by it is still of interest at all. It is this difference that carries the communicative Meaning ‘true’—for us. Digitally, in the strict operativity of numbers, this difference makes no sense. Virtually, everything that is possible is also real. What the truth then still Means is a question for us, the human observers. Meaning ‘truth’ has no equivalent in the mathematical-digital worlds beyond rule-based correctness. The question is therefore not whether it is possible to reach a consensus about what is true—that has never been possible and has therefore never been the decisive problem. Rather, the issue is whether there is still a consensus on the relevance of this question. In the digital society, trying to answer this question by measuring and calculating will not produce the truth, but at best “correct” signs. Truth loses its *Meaning* when it is not about facta or ficta, but about data, i.e., what works.

## 7 Digital ‘Bildung’

### 7.1 Digitization, digitalization and AI: the productive meaning of meaninglessness

During of its evolution, society creates within itself, with its own resources—this are signs—an environment free of communication, the expansion and meaningfulness of which it regulates itself along various connection possibilities—communication, information, music, visual art. By excommunicating certain signs—this is digitization—, society creates communication-free areas that can still be meaningfully labeled. Practices of counting and calculating offer society the possibility of productively dealing with the problem of external reference through self-reference, without thereby renouncing social consensus through the binding of perception. A conception of the world that is remote from communication, free of meaning, but not pointless—this is digitalization. It is the pure self-referentiality of the sense of numbers that enables a causal operativity of sign concatenation, i.e., a self-acting context of meaning. Their socially productive meaning lies precisely in their meaninglessness.

Understanding of digitization and AI is crucial: meaningful sign process and causal relationship are not contradictory (Kurzweil, 2024; Jin and Rinard, 2023; Metzinger, 2021). Contingency as a condition of sense does not imply the absence of causes, but rather their determinate indeterminacy. The contingency necessary for meaning processes arises from the overdetermination of each event. This enables an observer to decide which chain of causes to assign relevance to by distinguishing them from other possible causes: this is meaningful perception. Sense-making does not change the causal relationships, but is a sign process and precisely therein contingent, i.e., dependent and indeterminate at the same time. The observing system reacts contingently to a difference, i.e., always differently and yet causally, if it is automatically complex. Both the neuronal and the digital operativity of the sign process of sense-making are causal information contexts. In this way, intelligent sense systems are also possible that, like organizations or AI, manage without consciousness, understanding and intentionality in the sense of a will (Manhart and Wendt, 2021; Manhart, 2025b). In digitalization, the structural update of society is partially automated without human perception or consciousness still being significantly involved. The ‘humanistic’ fixation on communication and consciousness is therefore an obstacle to understanding modern society, which also includes the informatic self-generation of its (digital) environment. It also makes it difficult to adequately assess the possibilities of artificial intelligence: the environment of modern society communicates just as little as the structures of society are based alone on communication. This is especially true when an AI speaks. It does not understand, it does not communicate—it informs.

The fact that AI will have a major influence on the development of human individuals despite everything, or rather precisely because of this, is demonstrated not only by its successful use in schools and universities (Shin et al., 2025; Wang and Fan, 2025). Information-processing intelligence marks a fundamental difference to any conventional technology, but it is just another element in the ongoing co-evolution of humans with natural and social automats. This co-evolution is happening less in educational institutions than in everyday digital life. As a sign process, every AI automatically generates Sense (Manhart, 2025b). If we start from the ancient Greek *automaton*, then it means something that moves by itself, nothing more. We are not necessarily talking about technology or machines here. Rather, it is about dynamics that run on their own without human intervention (Manhart, 2024). Operational differences have nothing to do with the term *automaton*. Automata can be differentiated according to what is automated in them. A mechanical automaton is a self-acting machine that always produces the same output in response to inputs, but which executes the necessary intermediate steps without further intervention. This type of automation can be a car, a vending machine or a computer. However, this description does not apply to an adaptive biological or digital intelligence. In this case, automation does not concern mechanical power connections, but the concrete forms of internal dynamics themselves. What is automated here, on a very different operational basis, is information processing as a complex unit of structure-determined and structure-determining sense production: the learning (Manhart, 2019a,b; Manhart, 2025a,b).

Artificial intelligence is the automatic organization of electronic context structures and the adaptive reaction to a self-generated data construct *of oneself and the environment*. AI can use sensors to generate information about its environment, which it can process and learn from. It finds solutions to problems on its own and is therefore creative in this sense: it can compose, draw, speak, generate texts and calculate. All of this is possible even though it does not understand what it is doing. This non-understanding appears to many human observers as a considerable deficit, because human thinking in the mode of self-consciousness observes its own connectivity as understanding and emotionally values it as cognitive self-efficacy. On the other hand, this omission obviously does not change the intelligence of AI. Apparently, it is not necessary to understand, i.e., to process a self-observing consciousness, when composing a song, painting a picture or writing a text. This leads to considerable irritation on the part of human observers: humans cannot understand that the AI does not understand, even though they understand what it is saying.

### 7.2 Old question, new answers: know thyself in the digital age

In this irritation, however, there are considerable opportunities for insight and a new form of understanding ourselves, if not better, then at least differently. It could start with admitting that when AI speaks, we understand the sentences it says, but not the AI. The fact that both are possible at the same time corresponds to our everyday experience, that we understand what another person is saying, but we do not therefore understand this person. Of course, the difference to AI is much more fundamental. The different informatic operativity of AI also deprives a comprehensive concept of understanding, in the Meaning of bodily comprehension, of any basis. If we cannot achieve this with animals, which are organic beings (Nagel, 2016), then there is no hope at all with digital intelligence. Conversely, this also means that because of this operational difference, artificial intelligence, however intelligent and self-aware it may be, will not be able to understand what it is like to be human. Whether it even tries to do so and whether this helps humans or can even be useful is a completely different question. In any case, animals have rarely benefited from the fact, that we do not understand them, despite all our intelligence and intentions.

The co-evolution between humans and artificial intelligence has begun. The capabilities of AI are accompanied by responsive actor qualities for other participants in society that generate completely new social effects and have little to do with mechanical machine technology (Manhart, 2023a; Manhart and Wendt, 2024). The resulting hybrid ‘Bildung’ harbors possibilities and dangers whose scope is not yet foreseeable. From a phylogenetic perspective, there is only one more player in the co-evolution of humans with the natural and social automata of the past. If we broaden the horizon, however, what is co-evolving is not just humans, but other forms of intelligence and perhaps at some point also other forms of consciousness and understanding. The ideas that humans have of themselves, the characteristics and abilities that they ascribe to themselves, that they consider important and want to develop further, will once again change fundamentally in this co-evolution with artificial intelligence. Every behavior, ability or skill that was previously considered typically human and that an AI also exhibits will then point to the question of whether humans understand themselves. Dealing productively with this change in human self-understanding is the central task of an individual in the future society: digital ‘Bildung’.

## Data Availability

The original contributions presented in the study are included in the article/supplementary material, further inquiries can be directed to the corresponding author.

## Footnotes

^1^All German quotations in the text were translated into English by the author.
